# Face processing improvements in prosopagnosia: successes and failures over the last 50 years

**DOI:** 10.3389/fnhum.2014.00561

**Published:** 2014-08-05

**Authors:** Joseph M. DeGutis, Christopher Chiu, Mallory E. Grosso, Sarah Cohan

**Affiliations:** ^1^Boston Attention and Learning Laboratory, VA Boston Healthcare SystemJamaica Plain, MA, USA; ^2^Vision Sciences Laboratory, Department of Psychology, Harvard UniversityCambridge, MA, USA

**Keywords:** acquired prosopagnosia, developmental prosopagnosia, recovery, rehabilitation, treatment, cognitive training

## Abstract

Clinicians and researchers have widely believed that face processing cannot be improved in prosopagnosia. Though more than a dozen reported studies have attempted to enhance face processing in prosopagnosics over the last 50 years, evidence for effective treatment approaches has only begun to emerge. Here, we review the current literature on spontaneous recovery in acquired prosopagnosia (AP), as well as treatment attempts in acquired and developmental prosopagnosia (DP), differentiating between compensatory and remedial approaches. We find that for AP, rather than remedial methods, strategic compensatory training such as verbalizing distinctive facial features has shown to be the most effective approach (despite limited evidence of generalization). In children with DP, compensatory training has also shown some effectiveness. In adults with DP, two recent larger-scale studies, one using remedial training and another administering oxytocin, have demonstrated group-level improvements and evidence of generalization. These results suggest that DPs, perhaps because of their more intact face processing infrastructure, may benefit more from treatments targeting face processing than APs.

## Introduction

Prosopagnosia is a deficit in the ability to perceive and recognize faces, and most commonly results from genetic/developmental causes (up to 1 in 40 developmental prosopagnosics in the general population, Kennerknecht et al., 2006, 2008). More rarely, prosopagnosia is caused by acquired brain injury that damages occipital-temporal or anterior temporal regions (Barton, 2008). Though developmental and acquired prosopagnosics may have more or less severe perceptual deficits, they all generally have difficulties with building a rich holistic face representation sufficient for face identification (Bukach et al., 2006; Ramon et al., 2010; Avidan et al., 2011; Palermo et al., 2011; DeGutis et al., 2012b). Instead, prosopagnosics attempt to learn and recognize faces using a less effective piecemeal approach, or rely on non-facial cues such as voice and clothing. Reliance on these alternative methods leaves prosopagnosics with significant recognition deficits that may lead to a restricted social circle, more limited employment opportunities, and loss of self-confidence (Yardley et al., 2008). Because of these potentially debilitating consequences and the high prevalence of prosopagnosia, developing treatments to enhance face recognition is a valuable endeavor.

A widely held belief by clinicians and researchers is that prosopagnosics cannot significantly improve their face processing ability. Even as recent as 2005, Coltheart suggested that “there may be domains of cognition for which an impairment caused by brain damage is such that restoration of normal processing is impossible. It is conceivable that face processing is one such domain.” Coltheart goes on to suggest that this may be because “face processing depends on a specific brain region and this region may have a particular kind of structure that is specialized for the specific types of computations needed for recognizing the unique stimulus that faces are” (Coltheart et al., 2005). The acquired prosopagnosia (AP) literature somewhat reinforces Coltheart's claim, though more recent studies of developmental prosopagnosia (DP) (including two from Coltheart's group: Brunsdon et al., 2006; Schmalzl et al., 2008) suggest that improvement in some aspects of face processing, even at the group level, is indeed possible. In the current article, we first review the AP recovery and treatment literature and consider explanations of limited treatment-related improvements. We then review the more promising treatment-related improvements observed in DPs and discuss explanations for differences between developmental and acquired prosopagnosics.

## Method of search and selection criteria

Using pubmed, google scholar, and web of science as search engines, we searched for articles using the keyword “prosopagnosia” in conjunction with each of the following keywords: “recovery,” “training,” “treatment,” “therapy,” “rehabilitation,” “improvement,” “enhancement,” “amelioration,” “restoration,” and “compensation.” We included both peer-reviewed empirical articles and book chapters and focused our search on prosopagnosia due to acquired brain injury and DP (which includes congenital prosopagnosia). However, we excluded studies where prosopagnosia was a symptom of a more global deficit such as in cases of neurodegenerative disease (Cronin-Golomb et al., 2000; Turan et al., 2013) and autism spectrum disorder (Weigelt et al., 2012).

## Spontaneous recovery in acquired prosopagnosia

**Figure 1:**
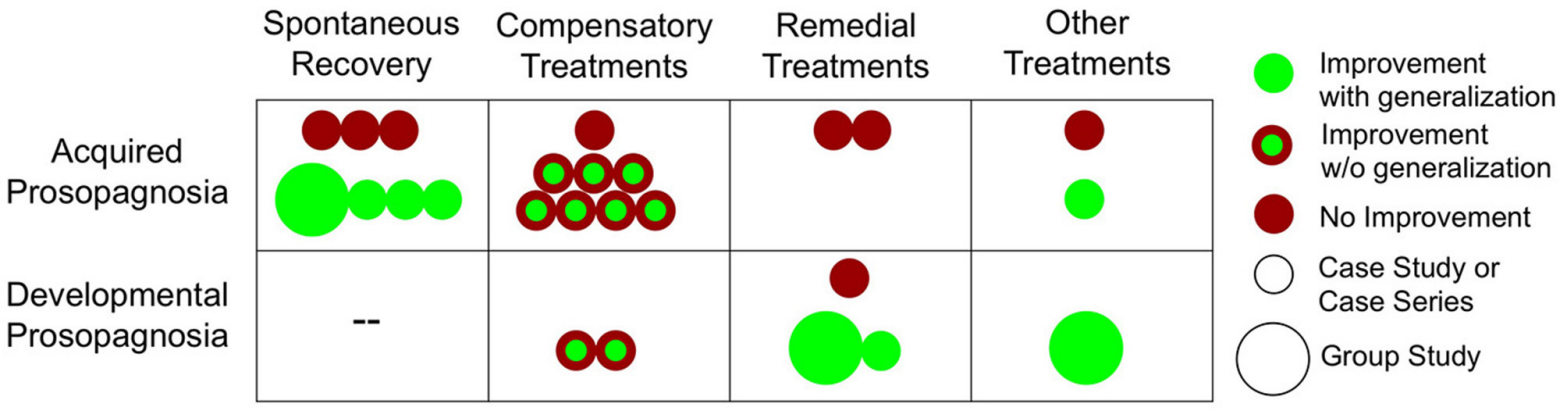
**Face processing improvements in acquired and developmental prosopagnosia**. For treatment studies, we defined generalization as improvements in face processing task(s) that were different from the intervention itself. For spontaneous recovery studies, since the intervention was time, we considered any increases in performance as improvements with generalization.

Studies of spontaneous recovery in AP are useful in that they can help determine the potential for the face processing system to naturally improve after damage, and can shed light on the possibilities for treatment-related improvements. As can be seen in Table 1 and Figure 1, our search revealed seven studies that assessed spontaneous recovery in AP, four of which suggest that recovery of face recognition abilities is possible. The first study to report recovery is a case of a 20-year old man who experienced prosopagnosia after falling from a horse and suffering bilateral, though predominantly left-sided, occipital-temporal contusions (Glowic and Violon, 1981). Remarkably, from 4 months post-injury to 1 year, the patient reported a full recovery in his face processing abilities. Because no neuroimaging data is presented, unfortunately it is difficult to know if this recovery was due to healing of the peripheral vasculature and support structures (e.g., reduced inflammation) or reorganization of the brain. Lang et al. (2006) provide more convincing evidence of neural reorganization, reporting full recovery after 6 months in an 89 year-old prosopagnosic woman with damage to right occipital-temporal regions. Interestingly, a post-recovery functional MRI revealed exclusive activation of the left fusiform face area (FFA) rather than the more typical right FFA activation when viewing faces, suggesting possible reorganization of face processing to homologous regions in the left hemisphere. Though these cases of full recovery are notable, they are somewhat limited by their reliance on the patients' self-report.

**Table 1:** **Spontaneous recovery in acquired prosopagnosia**.

**Source**	**Patient/N**	**Lesion location**	**Testing post-injury**	**Outcome**	**Improvements**
Glowic and Violon, 1981	Jean 20-year-old male	Bilateral occipital temporal, predominately left	T1: 4 months	Prosopagnosia abated according to self-report	Yes
			T2: 1 year 4 months		
Malone et al., 1982	1: 64-year-old male	1: bilateral occipital	T1: 10 weeks	1: Improved at recognizing familiar faces but not unfamiliar	Yes
	2: 26-year-old male	2: bilateral occiptal and right parietal	T2: 22 weeks		
			T1: 1 week	2: Improved at recognizing unfamiliar faces but not familiar	
			T2: 6 weeks		
Hier et al., 1983	*N* = 19	Lesion overlap: right temporal parietal	Examined at 2–4 week intervals until lost to follow-up	Projected recovery using life table chart: 50% recover after 9 weeks post stroke, 90% recover after 20 weeks	Yes
Sparr et al., 1991	H.C. 22-year-old female	Bilateral occipital	T1: 2 weeks	Face identification was poor when asked to identify photographs of well-known people (50%), primarily recognized people through prominent features	No
			T2: 40 years		
Ogden, 1993	M.H. 24-year-old male	Bilateral medial occipital	T1: 2 months	No improvement in prosopagnosia: impaired on discriminating age, gender, and expressions, of both familiar and unfamiliar faces, and primarily used features for recognition	No
			T2: 6 years and 2 months		
Spillmann et al., 2000	W.L. 73-year-old male	Bilateral medial parietal and medial temporo-occiptal	T1: 15 months	Face identification was still impaired due to a deficit in hollistic processing (could correctly identify and perceive all features but cannot efficiently integrate them)	No
			T2: 3 years 15 months		
Lang et al., 2006	89-year-old female	Right temporal-occipital	T1: N/A	Face recognition gradually returned with activation of the left fusiform face area	Yes
			T2: 6 months		

*T1, First testing session at specified time after injury; T2, Final testing session after injury*.

When using more objective tests of face perception and memory, Malone and colleagues described partial recovery in two acquired prosopagnosic patients with bilateral occipital lesions (Malone et al., 1982). One patient (64-year-old male) who was first assessed 10 weeks after symptom onset and again 12 weeks later, demonstrated improved recognition of familiar faces though not on perceptual discrimination of unfamiliar faces. Another AP (26-year-old male) was first assessed for prosopagnosia 1 week after an acquired brain injury due to a gunshot wound, and again 6 weeks post-surgery. He showed improved perceptual discrimination but no improvement on familiar face recognition. These two cases suggest that even with relatively similar lesions, the recovery of face perception and face memory mechanisms are dissociable and may represent two distinct targets for treatments.

In a fairly large group study of right hemisphere stroke survivors, Hier et al. (1983) reported that of 19 right hemisphere stroke patients suffering from prosopagnosia (according to performance on a famous faces test), 50% recovered after 9 weeks and 90% recovered after 20 weeks. Despite the relatively large number of patients in this study, a major limitation is that it relied exclusively on a famous faces test for diagnosis and tracking of prosopagnosia. Because they did not account for pre-morbid familiarity, this may have inflated the incidence of prosopagnosia and, because of potential practice effects, exaggerated the degree of natural recovery. An additional issue is that the group lesion overlap was centered in the temporal-parietal junction, which is significantly superior to occipital-temporal lesions typically associated with AP. Thus, these high recovery rates may not generalize to more typical cases of AP.

In contrast to these four studies showing evidence of recovery, three studies of patients with bilateral occipital-temporal lesions failed to find evidence of recovery. Comparing assessments 2 weeks after brain injury in a 22-year-old prosopagnosic, to assessments 40 years later, Sparr et al. (1991) did not find any evidence of recovery on an informal famous faces task. Ogden (1993) similarly failed to find evidence of any improvements of face processing functions in her study of a 24-year-old AP who was first tested about 2 months after injury and then 6 years post-injury. Finally, Spillmann et al. (2000) assessed their patient 15 months after stroke and then 3 years later with similar results of no recovery.

Collectively, these studies provide evidence that some recovery from AP is possible in certain patients. Considering the positive results of the patients with unilateral lesions (Glowic and Violon, 1981; Hier et al., 1983; Lang et al., 2006) along with the lack of recovery in patients with bilateral occipital-temporal damage (Ogden, 1993; Spillmann et al., 2000), it seems that unilateral lesions may have the best prognosis for recovery. Bilateral lesions likely damage homologous core face processing regions such as the occipital face area (OFA), FFA and the posterior temporal sulcus (pSTS) (Haxby et al., 2001), which may destroy key nodes in the face processing network (see more on this below). This is consistent with the observation that APs with bilateral damage have generally more severe face recognition deficits than those with unilateral damage (Barton, 2008). We did not find that recovery varied by age, gender, or handedness. Additionally, although it is likely that there is a graded window of recovery for AP that is similar to other acquired visual disorders (Zihl, 2011), besides the Hier study we did not find strong evidence that those initially assessed earlier showed more recovery. However, due to the small number of studies, variability across studies in methods of prosopagnosia diagnosis and time points used to assess recovery, the conclusions we can draw are limited.

In spite of these limitations, these studies suggest that the face processing system may have some capacity for neural reorganization after damage and leave open the possibility that treatments could significantly enhance face processing, potentially more for APs with unilateral lesions.

## Compensatory treatment approaches in acquired prosopagnosia

Several compensatory treatment attempts have been made to try to alleviate symptoms in AP, as seen in Table 2 and Figure 1. These treatments seek to teach patients ways to work around their face recognition deficits, either by using intact systems in the domain of perceptual face processing (e.g., attending to facial features), semantic processing (e.g., encoding a faces in conjunction with details about their profession), using verbal strategies (e.g., verbalize distinct facial features), or using intact implicit face recognition mechanisms. About half of these studies show some benefits (Beyn and Knyazeva, 1962; Polster and Rapcsak, 1996; Francis et al., 2002; Mayer and Rossion, 2007), though it is still an open question how much these treatments generalize^1^ beyond the faces used in the specific training programs.

**Table 2:** **Treatment approaches in acquired prosopagnosia**.

**Source**	**Patient/N**	**Lesion location**	**Compensatory/ remedial/ other**	**Duration of Treatment**	**Treatment**	**Outcome**	**Improvements**
Beyn and Knyazeva, 1962	C.H. 39-year-old male	Unknown	Compensatory	11 months	Systematic practice with facial expressions and facial features as well as practice copying faces	Self-reported improvement on recognition of faces and facial expressions	Yes without generalization
Wilson, 1987	O.E. 27-year-old male	Right temporal parietal	Compensatory	~3 weeks	Practice on facial recognition using visual imagery and motor movements	No significant improvements	No
Sergent and Poncet, 1990	P.V. 56-year-old female	Left anterior temporal and right temporal parietal regions	Compensatory	One session	Series of tasks that used semantic information to activate implicit face memories	Could overtly recognize faces when certain semantic information was given	Yes without generalization
De Haan et al., 1991	P.H. ~23-year-old male	Bilateral inferior occipital temporal	Compensatory	(1) One session	Using covert recognition to elicit overt recognition through (1) repeated exposure to familiar famous faces and (2) presenting occupational categories of faces	(1) No improvement	Yes without generalization
				(2) 3 sessions: pre-test, post-test immediately after and 2 months later		(2) Improvement on one out of six categories	
Polster and Rapcsak, 1996	R.J. 68-year-old male	Right occipital temporal	Compensatory	~2 weeks	Using different encoding instructions: rating features, rating personality traits, using distinctive features, and attaching semantic information	Improvement from rating traits and attaching semantic information but performed at chance when the faces were in different orientations	Yes without generalization
Francis et al., 2002	N.E. 21-year-old female	Primarily right temporal, possibly bilateral	Compensatory	Study 1: unfamiliar faces: 14 days, 7 two-hour sessions;	Study 1: (a) facial features and semantic information combined into one mnemonic (b) name mnemonic (c) rehearsal of name and face; Study 2: (a) semantic information and name (b) name alone	Conditions that simultaneously targeted both prosopagnosic and semantic impairments were most effective in improving face recognition	Yes without generalization
				Study 2: 14 days, 5 two-hour sessions			
Mayer and Rossion, 2007	P.S. 52-year-old female	Right inferior occipital and left occipital temporal	Compensatory	4 months, 2 sessions per week	Training to attend to and verbalize the internal facial features of novel faces and faces of her students	Significant improvement on recognizing faces using internal features, subjective improvement and increased confidence.	Yes without generalization
Powell et al., 2008	W.J. 51-year-old male	Left occipital, left frontal, bilateral temporal lobes, and right occipital lobe	Compensatory	4 × 1 h sessions for each condition over 2 weeks	4 conditions: picture with name, caricature with name, picture with name and semantic information, orienting attention toward distinctive features	Face recognition was significantly better when orienting to distinctive features, though not other conditions	Yes without generalization
Ellis and Young, 1988	K.D. 8-year-old female	Diffuse damage	Remedial	Over a period of 18 months	Discriminating familiar/unfamiliar/ schematic faces with feedback, learning face-name pairs with feedback	No evidence of improvement	No
DeGutis et al., 2013	C.C. 46-year-old female	Right occipital-temporal lobe	Remedial	30 sessions over 1 month	Training to integrate spacing information from the mouth and eye regions	Some improvement on training task but no generalization to novel face tasks	No
Wilkinson et al., 2005	R.C. 61-year-old male	Right temporal lobe, inferior frontal gyrus, superior parietal lobe	Other	4 × 1 h sessions	Administered galvanic vestibular stimulation to right or left vestibular nerve while performing face discrimination. Switched polarity halfway through each session	Improvement on the face-matching task after switching polarity (either right to left stimulation or left to right)	Yes with generalization
Behrmann et al., 2005	S.M. 24-year-old male	Right anterior and posterior temporal	Other	31 sessions over 4 months	Greeble training program	Improvement with greeble recognition but decline in face recognition	No

*Generalization: Evidence of improvements in processing novel face stimuli that are different from the treatment intervention itself*.

The first reported attempt at enhancing face recognition in prosopagnosia was by Beyn and Knyazeva (1962) who presented a 39-year-old patient (C.H.) with severe deficits in recognizing familiar faces, likely from bilateral occipital-temporal damage. Through systematic practice of faces with special attention to facial features and expressions, as well as practice copying faces, Beyn reported that C.H. showed some improvements in recognizing faces in real-world circumstances. Although neither standardized methods of training nor objective tests were used, this study provides preliminary evidence that attending to specific facial features may be beneficial in lessening face processing deficits.

Mayer and Rossion (2007) also showed some improvements using feature training in prosopagnosic P.S., a 52-year-old patient with damage to the regions involving the left fusiform gyrus (encompassing the left FFA) and right inferior occipital gyrus (encompassing the right OFA). They had P.S. verbally analyze internal facial features, progressing from (1) faces with caricatured features, to (2) unknown adult faces, to (3) unknown faces of children, and finally to (4) children in P.S.'s kindergarten class. P.S. was first asked to sort each set of faces based on a criterion feature (e.g., length of the mouth) and then to describe the distinctive internal feature for each face in the set. This strategy was then applied to her kindergarten class, where she made index cards of every child's distinctive internal facial features. After 4 months of training (two sessions per week), she improved at recognition of pictures of her students and reported relying more on internal features. Moreover, she could confidently stay with her students outside the school environment, suggesting some real-world training-related improvements.

Francis et al. (2002) also found some evidence for improvement after compensatory training in a 21-year-old (N.E.) with prosopagnosia and person-based semantic deficits due to primarily right, possibly bilateral, temporal lobe damage from herpes encephalitis. When comparing several compensatory face learning strategies, they found that the encoding approaches that targeted both semantic impairments and face processing deficits were the most effective—they not only improved recognition of unfamiliar faces, but also faces of individuals familiar to the patient. Despite these promising results, the authors caution that N.E.'s face perception abilities were largely intact and the improvements they observed may not hold for acquired prosopagnosics with more severe perceptual deficits.

Powell et al. (2008) also showed some face recognition improvement after providing different encoding strategies to acquired prosopagnosic W.J., who had damage to left occipital, left frontal, bilateral temporal, and right occipital regions (McNeil and Warrington, 1993; Powell et al., 2008). Compared to being provided with semantic information along with the faces or encoding faces with caricatured features, instructing the patient to attend to distinctive features (e.g., This is Victoria, she has large eyes and freckles) improved facial recognition the most. This provides additional evidence that attending to distinctive features can be a useful compensatory aid to face learning in APs.

Though these studies reported evidence of improvements and positive impacts on everyday life, other studies using compensatory feature and semantic training in APs have found very limited improvements (Polster and Rapcsak, 1996) or failed to find any improvements (Wilson, 1987). In a 68-year-old AP male (R.J.) with a right occipital-temporal damage and semantic impairments, Polster and Rapcsak (1996) compared several encoding instructions while R.J. attempted to learn new faces, shown from front-views. Between rating features (e.g., narrow-set vs. wide-set eyes), rating personality traits (e.g., lively vs. dull), identifying a distinctive feature (e.g., verbalize most distinctive feature), and attaching semantic information, encoding by rating personality traits and attaching semantic information yielded the most improvements during recognition of the same front-view versions of the faces. Unfortunately, these improvements did not generalize to improvements at recognizing novel ¾ views of these faces, suggesting that the information being learned was view-specific and may be of limited use in real-world settings. In another discouraging attempt, Wilson (1987) had a 27-year-old prosopagnosic with right temporal-parietal damage practice face recognition by attaching concrete visual images to each face and miming the image (e.g., This face is Sue—think of “soup” and mime eating soup). On each of the 11 test assessment sessions, performance did not demonstrate any appreciable improvement with either strategy.

Another compensatory approach with somewhat discouraging outcomes is the use of covert face recognition abilities, shown to be intact in some APs (though not all APs, see Barton et al., 2001), to improve overt recognition (i.e., provoked overt recognition). According to Burton's interaction and competition model of face recognition (Burton et al., 1990), covert recognition in APs arises from weak connectivity between face recognition units and person identity nodes (PINs), resulting in less activation of the PINs. The logic is that by incorporating semantic information (e.g., an individual's profession) while seeing someone's face, the activation of the PINs necessary for overt recognition could be strengthened, leading to improved recognition in APs. For example, Sergent and Poncet (1990) showed eight faces of famous politicians to acquired prosopagnosic P.V., who had damage to left anterior temporal and right temporal parietal regions. Though P.V. was unable to identify the faces, once the experimenter said that they all had the same occupation, she correctly guessed they were politicians and was able to identify seven out of eight faces. De Haan et al. (1991) replicated this effect in a limited way in a 23-year-old patient (P.H.) using a slightly modified paradigm in which the experimenters provided the category of profession. Out of the six categories they tried, improvements were limited to a single category in which the faces were highly related (actors from a particular soap opera). P.H.'s ability to recognize these faces faded after 2 months. Though using covert recognition mechanisms to aid overt recognition is theoretically appealing and may be possible in particular situations for certain patients (for a review see Morrison et al., 2000), the findings have been too inconsistent to be useful for more general rehabilitation.

Together, the results of compensatory training attempts in APs provide hope, but also suggest that no single approach is appropriate for all APs. Even with the most generally successful approach of focusing on distinct facial features, there are cases where it failed to work or where the effects of training failed to generalize beyond the faces used in training. One issue with many of these studies is that they did not adequately measure generalization to different tasks and different faces. Incorporating these measures of generalization in future studies would be useful to better gauge the therapeutic benefits of these approaches. One interesting pattern that we observed is that compensatory treatments were *more* successful in patients with *bilateral* lesions (e.g., Mayer and Rossion, 2007; Powell et al., 2008) compared to those with *unilateral* lesions (e.g., Wilson, 1987; Polster and Rapcsak, 1996). This stands in contrast to the spontaneous recovery results above, and paradoxically suggests that those with more extensive lesions have more to benefit from compensatory approaches. Though this could be an anomaly from the small number of studies in this literature, it warrants further investigation.

In sum, the available evidence suggests that one should choose compensatory treatments that are specific to each AP's deficits (e.g., perceptual vs. more semantic deficits) and their residual abilities (e.g., ability to identify distinctive features or identify personality traits from faces) as well as use guidance from theoretical models of face recognition (Bruce and Young, 1986; Haxby et al., 2001). However, considering the variable results of this rather small literature, a thoughtful trial-and-error approach using several treatments may be the most successful method in implementing compensatory training with APs.

## Remedial treatment approaches in acquired prosopagnosia

While compensatory training utilizes strategies to work around prosopagnosics' face recognition deficits, remedial training directly targets prosopagnosics' underlying deficits (i.e. holistic face processing) to promote more normal patterns of face processing. Despite evidence that face processing abilities can improve through recovery and compensatory training in some APs, there is currently no evidence that treatment approaches that attempt to directly remediate face processing in APs are effective (see Table 2 and Figure 1).

Ellis and Young (1988) present a very thorough attempt to retrain face discrimination in an 8-year-old prosopagnosic child (K.D.) with diffuse brain damage caused by meningococcal meningitis. In particular, over an 18-month period, they provided K.D. with systematic face discrimination training and face-name learning with feedback. Their thought was that perhaps systematic practice with a finite set of faces in a controlled environment would improve some aspects of face processing. They found no evidence of improvements after either repeated discrimination of familiar and unfamiliar faces or discrimination of schematic faces that differed on one to four features. They also failed to find any evidence that K.D. could learn face-name pairs. A potential drawback to this study is that the daily intensity of training was relatively low (on average, K.D. performed ~10 trials/day) and training was not sufficiently adapted to K.D.'s ability level (i.e. there were no face tasks that she could successfully complete at the beginning of training). This likely made the training tasks quite frustrating and discouraging. Even after considering that K.D. may have had reduced motivation, this study still provides evidence that the face processing system, once damaged, is not easily remediated even in a young, plastic brain.

More recently, DeGutis et al. (2013) used a higher intensity holistic face training program (30 sessions x 900 trials/session over 1 month) in a 46-year-old acquired prosopagnosic (C.C.) with a right occipital-temporal lesion. In particular, C.C. trained on a task in which she had to integrate configural information from the eye and mouth region to accurately categorize computer-generated faces into one of two arbitrary categories (faces with higher eyebrows and lower mouths are category 1, whereas faces with lower eyebrows and higher mouths are category 2). The logic was that these face judgments would be strategic and slow at first, and then with practice become faster and more holistic. Despite showing some modest improvements on the training task, C.C. did not show any appreciable generalization to assessments using novel faces (DeGutis et al., 2013). Notably, a smaller dosage of the same training program (15 vs. 30 sessions) has recently shown to enhance aspects of face perception and subjective face recognition abilities in a group of developmental prosopagnosics (see below, DeGutis et al., 2014). The discrepancy between C.C.'s results and that of DPs could reflect that it is more difficult to remediate AP compared to DP, though additional attempts to remediate AP are necessary to confirm this. Together, these results show no evidence that approaches which attempt to remediate face processing in AP are successful.

## Other treatment approaches in acquired prosopagnosia

In addition to these compensatory and remedial approaches in AP, researchers have tried other means to improve face processing in APs. Wilkinson et al. (2005) used galvanic vestibular stimulation in a 61-year-old patient with AP from extensive damage to the right hemisphere, including the entire temporal lobe, inferior frontal gyrus, and superior parietal lobe. Their logic was that since face-selective brain regions are strongly activated by vestibular stimulation (Bense et al., 2001), electrical stimulation of the vestibular system may restore aspects of face perception. Electrical currents were administered via the left and right vestibular nerves during a forced choice face-matching task. Accuracy significantly improved from chance level to 70% after switching the stimulation polarity from either right to left or from left to right (Wilkinson et al., 2005). These improvements could be from generally enhancing alertness/attention or from the vestibular system's effects on visuospatial perception (Wilkinson et al., 2008).

Using a different approach, Behrmann et al. (2005) tried to improve face processing in an AP by training within-category discrimination of face-like objects (“greebles,” Gauthier and Tarr, 1997). Their logic was that greeble training would engage visual expertise mechanisms similar to that of faces, and that stimulating these expertise mechanisms may enhance face perception. In particular, 24-year-old acquired prosopagnosic patient S.M. who suffered damage to his right anterior and posterior temporal regions, was trained to become a greeble expert over a period of 31 sessions (at least two sessions per week). Although the patient demonstrated marked improvements with recognizing greebles, he showed *more* impairment in facial recognition post-training, suggesting some potential competition between greeble processing and face processing. This study makes the important point that in order for an acquired prosopagnosic to improve at face processing, they likely have to train with faces.

## Why do treatments produce rather limited improvements in acquired prosopagnosia?

Together, the AP recovery and rehabilitation literature is consistent with Coltheart's view that the capacity to restore face processing abilities to normal levels is limited. However, there is evidence that at least some recovery is possible and that compensatory treatments can produce improvements, though it remains to be determined if these improvements generalize and if these strategies will be useful tools for APs in their everyday lives.

One explanation for the limited capability to restore normal face processing in AP is, as Coltheart suggests (2005), because face processing relies on specific cognitive (e.g., holistic processing) and neural mechanisms (e.g., core face processing regions which include the FFA, occipital face area-OFA, and posterior superior temporal sulcus-pSTS). It could be that when these face-selective mechanisms are damaged, because of differences between face and object processing and the limits of neural plasticity, they cannot be taken over by more general object processing mechanisms. The existence of a double-dissociation between prosopagnosics with normal object processing and patients with impaired object processing but intact face processing (Moscovitch et al., 1997; Germine et al., 2011) supports this distinction between object and face processing. If face-specific neural mechanisms become damaged, it may be that more general object recognition mechanisms cannot be used to efficiently recognize faces, but possibly can only aid in more effortful feature processing. This would account for some of the success of compensatory training in which APs are taught to verbalize distinct features (e.g., Mayer and Rossion, 2007). The distinctiveness of face and object processing may also explain why training on face-like objects (greebles) failed to improve face processing.

Another explanation for limited treatment-related improvements in AP is that to some degree, face processing sub-regions in the core (FFA, OFA, pSTS) and extended networks (anterior temporal lobes) represent distinct, independent functions and are not redundant. This lack of redundancy within the face processing network could reduce the capacity for reorganization amongst intact regions and make it so that damaging any single region is more catastrophic. Evidence for specialization amongst face processing regions is from an fMRI study showing that the FFA is sensitive to both face parts and face configuration, while the OFA and pSTS are sensitive to the presence of real face parts but not to the correct configuration of those parts (Liu et al., 2010). Furthermore, the pSTS has shown to be much more sensitive to dynamic aspects of faces (e.g., facial expressions) than the FFA or OFA (Pitcher et al., 2011). Patient studies also support functional independence within the face processing network. Barton (2008) found that patients with lesions to right occipital-temporal regions had more specific deficits in perceiving facial structure and configuration, particularly of the eye region, whereas those with more anterior temporal damage had greater deficits in accessing face memories.

Though face regions may be highly specific within a hemisphere, there may be more redundancy across hemispheres (e.g., right and left FFA). This redundancy would go along with findings that unilateral lesions are typically associated with less pronounced deficits than bilateral lesions (unilateral: Barton, 2008; in contrast, bilateral: Rossion et al., 2003) and why more APs recover after unilateral lesions than bilateral lesions. Furthermore, some redundancy amongst homologous areas can help explain Lang et al.'s (2006) demonstration of complete recovery as well as engagement of the left FFA after damage to right occipital-temporal regions. Despite some redundancy, homologous regions might have somewhat different functional properties. For example, one functional imaging study has suggested that feature- or part-based face processing characterizes the function of the left FFA, while whole-face processing characterizes that of the right FFA (Rossion et al., 2000).

The differentiation between face and object processing, further specialization amongst face selective regions, and even specialization of face selective regions in each hemisphere, may combine to make face recognition particularly depend on coordination amongst nodes in a highly specific network. Indeed, evidence suggests that the coordination amongst face processing nodes may be a crucial aspect of successful face processing (Moeller et al., 2008). This specialization in a network may make it so that the function of a single face-selective region cannot be fully taken over for by the remaining face processing regions and clearly cannot be taken over by regions that represent non-face processing regions. The relative specificity of face processing contrasts with acquired brain injuries causing aphasia (i.e., dysfunction in language comprehension or expression), where evidence suggests that peri-lesional and homologous regions can take over functions of damaged regions (Hamilton et al., 2011; Shah et al., 2013). This may reflect more redundancy in language processing compared to face processing. This high level of specialization and expertise involved in face recognition may make it more vulnerable to disruption and result in AP having a somewhat limited capacity for treatment (for a more extensive discussion of neural plasticity in face processing and prosopagnosia, see Bate and Bennetts, 2014).

## Attempts to enhance face processing in developmental prosopagnosia

As can be seen in Table 3 and Figure 1, the current evidence suggests that compared to the AP findings there may be more potential for treatment-related face processing improvements in DP. In our review of the current literature, five out of six attempts with DP showed some degree of success in bettering aspects of face processing, three of which showed evidence of generalization beyond the faces used in training. It is also notable that there have been two recent group treatment studies (Bate et al., 2014; DeGutis et al., 2014). These studies are important in testing whether treatments work on a DP population level rather just for particular cases.

**Table 3:** **Treatment approaches in developmental prosopagnosia**.

**Source**	**Patient/N**	**Compensatory/ remedial/ other**	**Duration of Treatment**	**Treatment**	**Outcome**	**Improvements**
Brunsdon et al., 2006	A.L. 8-year-old male	Compensatory	~1 month	Using defining facial characteristics to learn faces of familiar people	Improvement on trained faces with and without hair and from different viewpoints, reported real-life improvements	Yes without generalization
Schmalzl et al., 2008	K. 4-year-old female	Compensatory	9 sessions over a month	Using defining facial characteristics to learn faces of familiar people	Immediately post-training improvement on front view recognition and more normal scan paths, 4 weeks after training also improved at recognition of faces from different viewpoints	Yes without generalization
DeGutis et al., 2007	M.Z. 48-year-old female	Remedial	~14 months	Training to integrate spacing information from the mouth and eye regions	Significant improvement on face perception and recognition, self-reported improvements, more face-selective N170 and enhanced fMRI connectivity with face-selective regions	Yes with generalization
Dalrymple et al., 2012	T.M. 12-year-old male	Remedial	47 sessions over 10 months	Practice on one face (mother's) with feedback	No significant improvements	No
DeGutis et al., 2014	*N* = 24	Remedial	15 sessions over 3 weeks	Training to integrate spacing information from the mouth and eye regions	Improvement on face perception, daily face recognition, and increased holistic processing in better trainees, no improvement of faces from varying viewpoints	Yes with generalization
Bate et al., 2014	*N* = 10	Other	2 sessions over 14–25 days	24 IU of intranasal oxytocin and placebo spray	Improvements on facial memory and face matching task for DPs but not controls.	Yes with generalization

*Generalization: Evidence of improvements in processing novel face stimuli that are different from the treatment intervention itself*.

## Compensatory treatment approaches in developmental prosopagnosia

Brunsdon et al. (2006) published the first positive attempt to rehabilitate an eight-year-old developmental prosopagnosic (AL) using “feature naming” training, a compensatory approach similar to those used in AP. In particular, AL was taught to perceive, discuss, and remember five distinctive facial characteristics of 17 faces of people he knew. The first two characteristics were always age and gender (which AL could likely recognize) and the other three characteristics were distinctive facial features such as “long thin face,” “wide nostrils,” “high curved eyebrows,” “wrinkles around the eyes,” and “freckles.” After 14 practice sessions over 1 month, AL showed improved recognition of not only the originally trained face images, but also of images of the same faces from different angles with and without hair. He also reported anecdotal real-life improvements of recognizing these faces.

Using the same training approach as Brunsdon et al. (2006), Schmalzl et al. (2008) showed similar positive results with 4-year-old developmental prosopagnosic K. K. not only showed improvements in recognizing target faces, but 4 weeks after training, she also improved on recognizing the faces in different orientations. Additionally, before training K. made abnormal eye movements focused on the external aspects of the face and after training, her scan paths were more normal and involved greater scanning of internal features. This more normal pattern of scanning internal features also generalized to untrained faces. Together, these results suggest that by training compensatory mechanisms in DP children, it is possible to enhance recognition of trained faces, and that this may lead to more normal face scanning patterns.

It is possible that these compensatory strategies could also help adult developmental prosopagnosics. Like K. before training (Schmalzl et al., 2008), adult DPs have shown to have more dispersed eye movements and more often fixate on external facial features (Schwarzer et al., 2007). Thus, similar compensatory training may result in adult DPs paying more attention to the internal features and better remembering particular faces. However, compensatory training could be less effective in adult DPs because they may be already quite well-practiced at using compensatory strategies, including attending to distinctive features.

## Remedial treatment approaches in developmental prosopagnosia

In addition to the positive results of compensatory training in children with DP, evidence suggests that remedial training in DPs can produce more general improvements in face processing (DeGutis et al., 2007, 2014). An advantage of this approach over compensatory approaches is that it is more automatically implemented, which may better promote generalization.

The training procedure used in two of these studies was very similar and targeted enhancing holistic face processing. The rationale was that DPs could apply some holistic processing to faces, but only over a spatially limited area (e.g., Barton et al., 2003; DeGutis et al., 2012b) and the aim of training was to enhance prosopagnosics' ability to perceive internal feature spacing information across a greater spatial extent of the face. To accomplish this aim, DeGutis et al. (2007) designed a task where participants make category judgments based on integrating two vertical feature spacings: the distance between the eye and eyebrows, and between the mouth and nose. It was thought that, after thousands of trials, DPs could learn to allocate attention to both feature spacings simultaneously, resulting in greater sensitivity to configural information across the inner components of the face (i.e., greater holistic processing).

The first study using this procedure had a 48-year-old DP (M.Z.) perform several months of this procedure (over 20,000 trials; DeGutis et al., 2007). After training, she showed improvements on standardized tests of face perception/recognition (e.g., Benton Face Perception Test) and also experienced daily life improvements. M.Z. reported that these effects lasted for several months before fading. Additionally, immediately following training, she demonstrated a more normal pattern of event-related potential selectivity, showing a greater N170 (an occipito-temporal potential normally selective to faces and thought to reflect holistic face processing, see Jacques and Rossion, 2009) in response to faces than objects, and enhanced functional MRI connectivity within right hemisphere face-selective regions during face viewing. These signatures of normal face processing were not present before training. This suggests that it is possible to enhance face recognition in an adult DP using a remedial approach and that this can enhance signatures of normal face processing.

A recent study of 24 DPs that used a similar procedure (though participants performed only 15 sessions of training rather than >50) suggests that face processing can be enhanced at the group level (DeGutis et al., 2014). After training, DPs demonstrated overall enhanced performance on several face perception tasks as well as evidence of daily life improvements on a self-report diary. Furthermore, those who particularly excelled at the training task showed the strongest improvements on measures of face perception and enhanced holistic face processing. In fact, whereas prior to training there was a marked difference in holistic face processing between better trainees and controls, after training there were no significant differences between the two groups. However, not all aspects of face processing were enhanced—there were no improvements on measures that required face discrimination from different viewpoints, tasks shown to be particularly challenging for prosopagnosics (Marotta et al., 2002; Lee et al., 2010).

In contrast to these positive reports of training holistic face processing in DPs, there is one report of a failed remedial attempt in an adolescent DP (Dalrymple et al., 2012), which used a somewhat similar training approach to Ellis and Young (1988). Dalrymple et al. (2012) reported an attempt by DeGutis and colleagues to train 12-year-old T.M. to recognize the face of his mother. T.M. made a “mom/not-mom” response when presented with a picture of either his mother or age-matched females, and was provided feedback after each response. After 47 sessions of training (~10–15 min per session) over a span of 10 months, T.M. did not demonstrate any appreciable improvements on the mom/not-mom task nor did he report improvements in daily life. Similar to Ellis and Young (1988), the intensity of training was somewhat low and insufficient motivation could have been a factor. Regardless, the results of this study are cautionary and suggest that there could be limitations to improvements in face processing in DPs even in the younger, developing brain.

Together, these studies suggest that remedial cognitive training that targets holistic face processing can enhance face processing in DPs and can potentially generalize to improvements in everyday life. Though remedial training did not help all DPs nor did it even enhance all aspects of face processing in the DPs it did help, these studies provide compelling evidence that the face processing system in DPs is at least partially remediable rather than permanently deficient.

## Other treatment approaches in developmental prosopagnosia

In addition to remedial training, another recent promising study by Bate et al. (2014) attempted to improve face processing in developmental prosopagnosics by administering intranasal oxytocin. Oxytocin is a neuropeptide that has shown to be involved is several aspects of social cognition including pair-bonding and trust (Walum et al., 2012) and may be dysfunctional in individuals with deficits in social cognition such as autism. Oxytocin has also shown to enhance the ability to infer the mental state of others on a task that requires sensitivity to subtle information from the eye region (Baron-Cohen et al., 2001; Domes et al., 2007). This is relevant to prosopagnosia in that the eye region is highly diagnostic for face recognition (Butler et al., 2010) and that processing of the eye region has been shown to be particularly impaired in prosopagnosics (DeGutis et al., 2012b). Further supporting this link between oxytocin and facial recognition ability, a recent study of 178 families with at least one autistic child found that variation in the oxytocin receptor gene, *OXTR*, was strongly associated with face recognition performance on the Warrington Face Memory Test (Skuse et al., 2014).

In light of these associations between oxytocin and facial recognition, Bate et al. (2014) attempted to enhance face perception and face memory in DPs using intranasal oxytocin. Ten DPs and ten normal controls were given both oxytocin and a placebo spray, with participants and experimenters both blind to condition assignment. Forty-five minutes after inhalation of the drug or placebo, participants completed novel versions of the Cambridge Face Memory Test (CFMT) and a simultaneous face-matching task. The results showed that DPs had significantly better performance on both tasks after inhaling oxytocin compared to when they inhaled placebo, while the control group showed no differences between conditions. DPs' improvement on both well-validated face memory and perception tasks is notable. Though the mechanisms of this improvement remain to be elucidated, one possibility is that oxytocin enhanced face-specific attention mechanisms, such as to internal features or the eye region in particular. These promising results suggest that further exploration of oxytocin's potential to produce longer-lasting improvements would be an exciting future direction not only for DPs, but for APs as well.

## How do treatments improve face processing more in DPs more than APs?

The studies reviewed above demonstrate that developmental prosopagnosics can benefit from several types of treatment. Thus, we suggest that compared to acquired prosopagnosics, developmental prosopagnosics may have a substantially greater capacity for improvement.

A likely explanation for DPs' potentially greater ability to benefit from treatments than APs is that they have a more intact face processing infrastructure compared to APs. Though studies have reported structural neural differences between DPs and controls (Behrmann et al., 2007; Garrido et al., 2009; Thomas et al., 2009), these differences are subtle when compared to the typically larger, more absolute lesions associated with AP (Barton, 2008). For example, Garrido et al. (2009) found that compared to controls, DPs had reduced cortical volume in the right anterior fusiform/temporal region, right middle fusiform gyrus, and superior temporal regions. They also found that better scores on face identity tasks were significantly correlated with the volume of the right middle fusiform gyrus. In addition to these cortical differences between DPs and controls, Thomas et al. (2009) report preliminary evidence that DPs have reduced white matter integrity between occipital-temporal and occipital-frontal regions, suggestive of compromised connectivity within the face processing network and between face processing regions and more anterior regions. Together, this suggests that despite not having gross anatomical differences from controls, DPs have subtle structural differences that likely contribute to their face recognition deficits. Though these subtle structural differences may be important aspects of DPs' face recognition deficits, their subtlety may allow for greater neural plasticity and treatment-related improvements compared to acquired prosopagnosics who may have more catastrophic structural damage (for additional discussion on neural plasticity in face processing with regards to prosopagnosia, see Bate and Bennetts, 2014).

In addition to having structure similar to controls, several recent studies provide evidence that DPs' face processing mechanisms are not qualitatively different from controls, but instead show more subtle quantitative differences. For example, DPs generally have a normal face selective N170 ERP component, which represents relatively normal earlier stages of perceptual processing, but have a reduced N170 difference between upright and inverted faces, which may reflect reduced holistic face processing or the use of somewhat similar mechanisms for upright and inverted faces (Towler et al., 2012). Notably, unlike DPs, the majority of individuals with AP do not show a face selective N170 (Dalrymple et al., 2011; Prieto et al., 2011), which may explain some of the differences in treatment success between APs and DPs. Additional ERP evidence for similarities between DPs and controls is that during successful face recognition, DPs show normal N250 and P600f ERP components, potentials related to early visual and later post-perceptual stages of face recognition. This suggests that on the rare occasions that DPs recognize a face, they use similar mechanisms as controls. Furthermore, in functional MRI scans, DPs have shown some face selectivity amongst the core face processing regions (Bentin et al., 2007; Minnebusch et al., 2009; Furl et al., 2011), albeit they may have fewer face selective regions and may show slightly reduced selectivity (Furl et al., 2011).

Together, these studies suggest that DPs may have the ability to process faces in a way that is qualitatively similar to controls, but may have disrupted connectivity within the face processing system. It could be that treatments are improving face recognition in DPs by boosting connectivity within DPs' intact face processing infrastructure. Evidence supporting this idea is from DeGutis et al. (2007) who found increased coherence amongst face-selective regions after training.

DPs' subtle differences from controls and capacity for improvement have interesting similarities and differences with other developmental disorders affecting face processing. For example, the lack of an N170 inversion effect is also found in autism and Williams Syndrome (Towler and Eimer, 2012). Additionally, both individuals with autism and those with DP show dysfunctional face adaptation effects (Pellicano et al., 2007; Palermo et al., 2011). This may suggest that these disorders share a common abnormal developmental trajectory. However, in contrast to autism and Williams Syndrome that are defined in part by marked social differences, DPs show more typical social behavior. For example, it has been shown that DPs attend to the eye region as much as healthy controls (DeGutis et al., 2012b), and that many can efficiently recognize emotion (Palermo et al., 2011; though see Le Grand et al., 2006) and gender (DeGutis et al., 2012a; though see Kress and Daum, 2003) from faces. Furthermore, evidence suggests that holistic face processing is a core deficit in DP (DeGutis et al., 2012b; as well as acquired prosopagnosia, see Busigny et al., 2014) while this is not the case with autism (see Weigelt et al., 2012 for a review) or Williams syndrome (Bellugi et al., 2000). Together, this suggests that unlike autism and Williams syndrome in which there are more global developmental consequences, DP is more specifically associated with developmental abnormalities in face processing. These abnormalities are more quantitatively than qualitatively different from controls.

Though the current DP treatment studies demonstrate that face processing improvements are possible from training, it still remains to be seen whether DPs can truly achieve normal face recognition abilities. Even in cases where treatments were effective at improving face processing (Bate et al., 2014; DeGutis et al., 2014), DPs' abilities either continued to be below average or the skills learned did not generalize to all aspects of face processing (e.g., did not generalize to discrimination across viewpoints in DeGutis et al., 2014). Furthermore, even after successful training, evidence suggests that skills may not be “self-perpetuating” (e.g., DeGutis et al., 2007) and it is likely that without continued intervention DPs return to their dysfunctional ways of perceiving and remembering faces. Thus, though the current demonstrations lay the groundwork for the treatment of DP, there is much work ahead to create effective long-lasting treatments (for additional discussion on future directions, please see Bate and Bennetts, 2014).

## Summary

Prosopagnosia has a high incidence (particularly DP) and can significantly impair social engagement and everyday functioning (Yardley et al., 2008). Currently there are no widely accepted treatments and instead, prosopagnosics are commonly left to learn how to recognize individuals through their own process of trial-and-error with alternative strategies (e.g., voice, gait, clothing, etc.). In our review of the literature, we find evidence that effective treatments are just beginning to emerge. Though the most consistent treatment successes have been in DP, we find some evidence for the capacity for improvements in AP as well. In addition to enhancing the daily functioning of prosopagnosics, understanding how to better improve face processing could also lead to helping several other populations with face processing and social cognitive deficits including those suffering from autism, Williams syndrome, schizophrenia, as well as those with age-related cognitive decline and dementia. Finally, understanding the mechanisms of these treatments and how successful treatment impacts the cognitive and neural signatures of face processing can lead to broader insights into the capacity for cognitive systems and the brain to reorganize.

### Conflict of interest statement

The authors declare that the research was conducted in the absence of any commercial or financial relationships that could be construed as a potential conflict of interest.

## Footnotes

^1^For the following treatment studies, we defined “generalization” as evidence of improvements in processing novel face stimuli that are different from the treatment intervention itself. Studies that do not have evidence of generalization by this definition could still have real-world implications (e.g., training to specifically better recognize a friend's face) but may be less useful than studies with evidence of enhancing more global aspects of face processing abilities.
